# When is a disparity not a disparity? Toward an old theory of three-dimensional vision

**DOI:** 10.1177/20416695231202726

**Published:** 2023-10-11

**Authors:** Brian Rogers

**Affiliations:** Experimental Psychology, University of Oxford, Oxford, UK

**Keywords:** optic flow, three-dimensional perception, binocular vision, contours/surfaces, cue combination, depth, perception, scene perception, stereopsis

## Abstract

The aims of this paper are twofold: first, to discuss and analyze the concept of binocular disparity and second, to contrast the traditional “air theory” of three-dimensional vision with the much older “ground theory,” first suggested by Ibn al-Haytham more than a thousand years ago. The origins of an “air theory” of perception can be traced back to Descartes and subsequently to the philosopher George Berkeley, who claimed that distance “could not be seen” because points lying along the same line of sight (in an empty space) would all project to the same location on the retina. However, Descartes was also aware that the angle of convergence of the two eyes could solve the problem of the “missing” information for the monocular observer and, since then, most visual scientists have assumed that eye vergence plays an important role both in judging absolute distance and for scaling retinal size and binocular disparities. In contrast, al-Haytham's and Gibson’s “ground theories,” which are based on the geometry of the textured ground plane surface that has surrounded us throughout evolution and during our lifetimes, are not just more ecologically based but they also obviate the need for *disparity scaling*.

## Background

**Figure 1.**
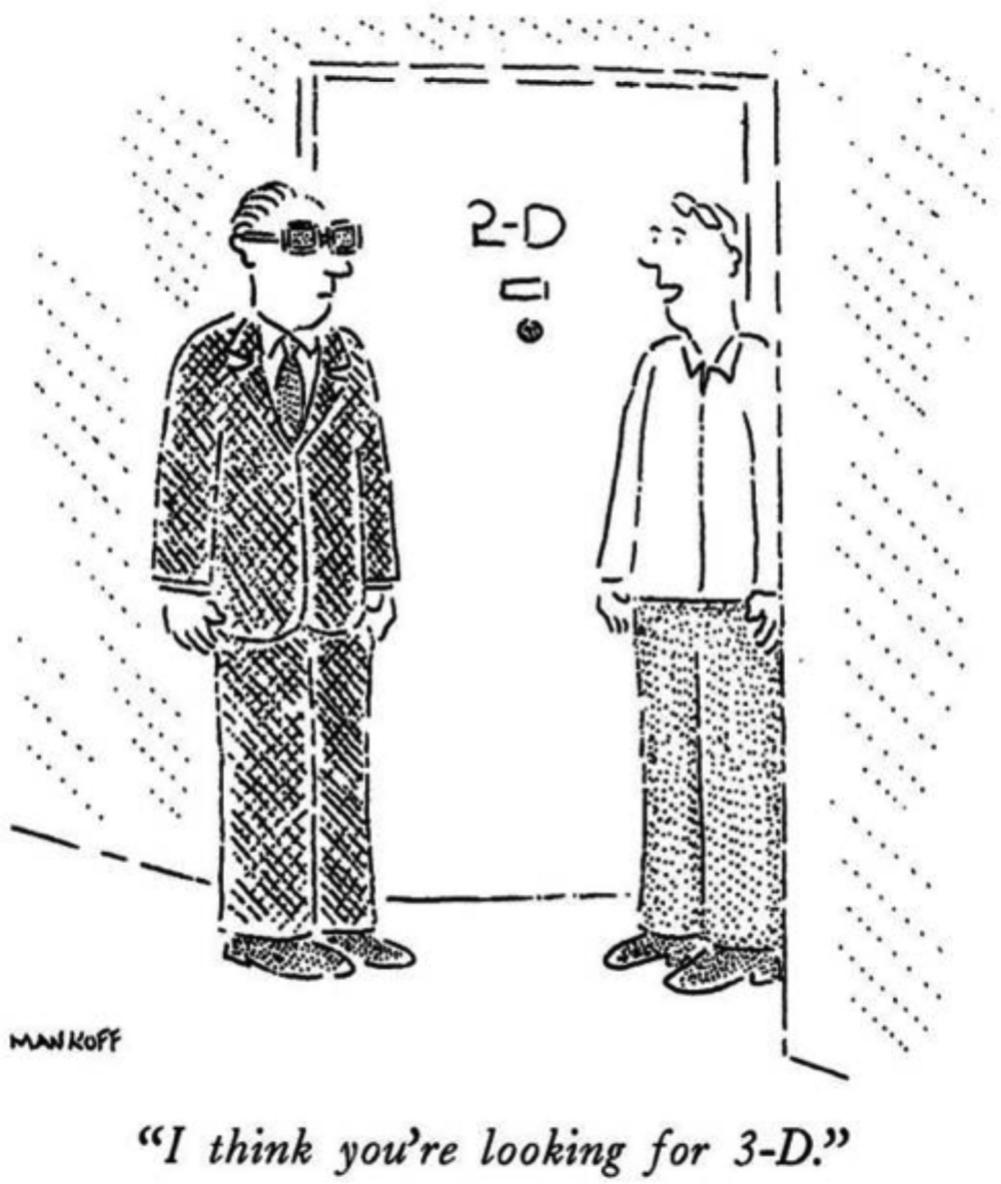
From the *New Yorker* March 7th, 1988.

**Figure 2.**
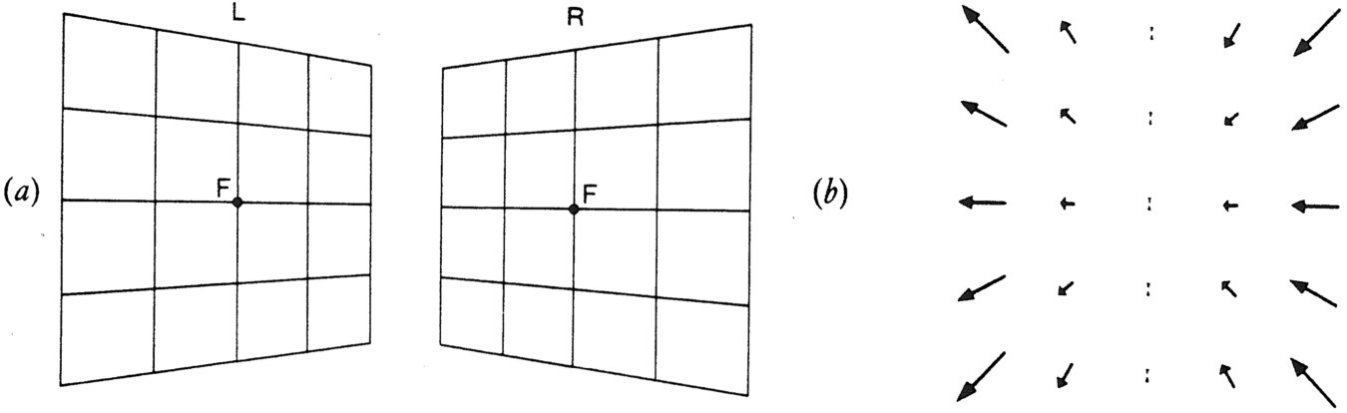
(a) The projection of a frontal surface that is close to the observer's two eyes onto two flat planes. (b) Note that the differences or disparities between the two eyes’ images are in both a horizontal and a vertical direction, as shown by the arrows (reproduced from Tyler, 1991a).

The concept of binocular disparity is well-known but not necessarily correctly understood (Figure 1). Most textbook descriptions refer to binocular disparities in terms of the differences between the two retinal images, as well as noting that the majority of the differences are “horizontal,” as a consequence of the “horizontal” separation of the two eyes (e.g., Bruce et al., 2003). As a result of the mathematical analyses of Mayhew and Longuet-Higgins (1982) and Gillam and Lawergren (1983), attention has also been drawn to the vertical differences between the retinal images—the so-called “vertical disparities” (Howard & Rogers, 1995; Rogers & Bradshaw, 1993). A complicating factor is that the disparity field is often depicted in terms of the projections of the world onto two flat planes (Figure 2), rather than the differences between the optic arrays, or the retinal images they create. Whilst this is of only minor importance, because those projections can easily be mapped onto any other surface, flat plane projections have the disadvantage that they are not able to capture the characteristics of the large visual fields that we actually experience in real-world perception (Johansson & Borjesson, 1989).

However, identifying the characteristics of the retinal images becomes important when it comes to thinking about how the human (or other) visual system might extract those differences—the disparities—between the two eyes’ retinal images (Frisby, 2009; Rogers, 2009). But why is it necessary to use quotation marks around the words “horizontal” and “vertical”? If we are considering the retinal images, the terms horizontal and vertical might refer to axes of coordinate systems located in the two eyes (oculocentric coordinates) or with respect to the head (headcentric coordinates; Howard, 1982) but both possibilities suffer from the problem that whenever the eyes or head are rotated around the near-far (*z*) axis, “horizontal” is no longer horizontal and “vertical” is no longer vertical.

## Corresponding Points and the Vieth-Müller Circle

**Figure 3.**
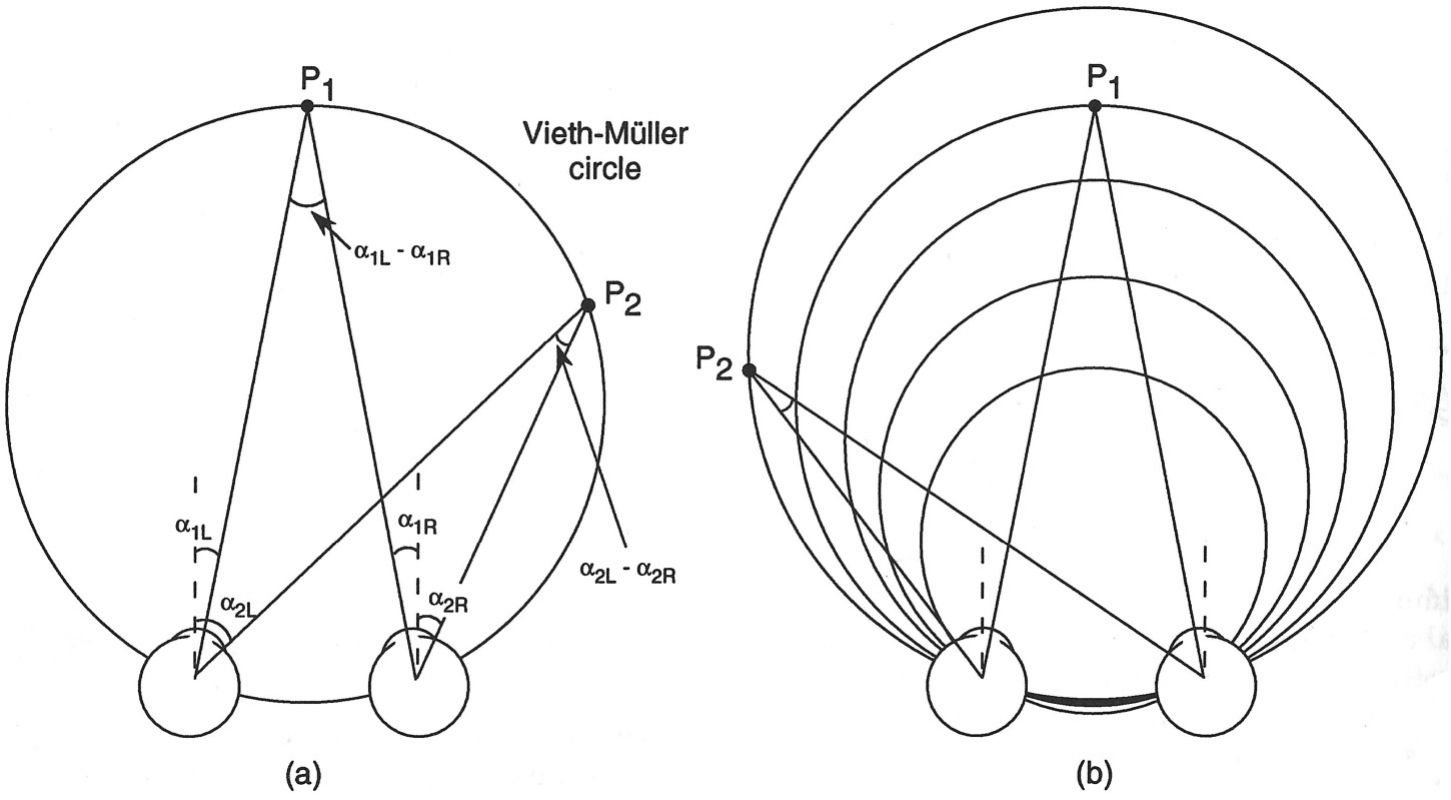
(a) The Vieth-Müller (V-M) or iso-vergence circle is the locus of points in the world that stimulate *corresponding points* in the images presented to the two eyes. Corresponding points, by definition, have zero disparity. (b) The diameter of the V-M circle varies as a function of the vergence distance (Howard & Rogers, 1995).

Traditional geometric analyses of binocular vision are based on the notion of *corresponding points* in the two eyes—that is, anatomically identical locations on the two retinal surfaces (Figure 3a). For example, the Vieth-Müller (V-M) circle describes the circular locus of points in the *plane of regard* (i.e., the plane that passes through the nodal points of the two eyes and the fixation point) that stimulate corresponding points in the retinal images of the two eyes (assuming appropriate vergence and the torsional alignment of the eyes). The points on the V-M circle can also be described as the points of equal vergence of the two eyes if the eyes are fixated to the left or to the right of the median plane (Figure 3b). Note that the radius of the V-M circle varies inversely with the angle of convergence of the two eyes. This particular locus of points is referred to as a *geometric* (as opposed to an empirical) *horopter* (Tyler, 1991a).

The question my students often ask me is whether the V-M circle has any significance other than being a fact of geometry. Inspection of Figure 3a and b shows that for a series of points (or a surface) that curves around a particular V-M circle, those points (i) do not lie in a *frontal plane* or any other flat surface and (ii) nor are they *equidistant* from the observer—two properties that would be useful to an observer. Moreover, although the stimulation of anatomically corresponding points provides the information that those points (or that surface) have a *curved* locus in space, they tell us nothing about the degree of curvature (or flatness) because that depends on the vergence distance. In other words, the stimulation of anatomically corresponding points in the two eyes tells us rather little about the 3D structure of surfaces, objects, and their layout in the surrounding world. The same point can be made about all *geometric* horopters^1^—geometric horopters describe the geometry of the situation but tell us rather little about the 3D characteristics of the world we perceive. On the other hand, the identification of *empirical* horopters, for example, whether a surface *appears* to be flat, curved, or equidistant, has been extremely useful in revealing the characteristics of the underlying binocular mechanisms.

## The Measurement of Visual Direction

**Figure 4.**
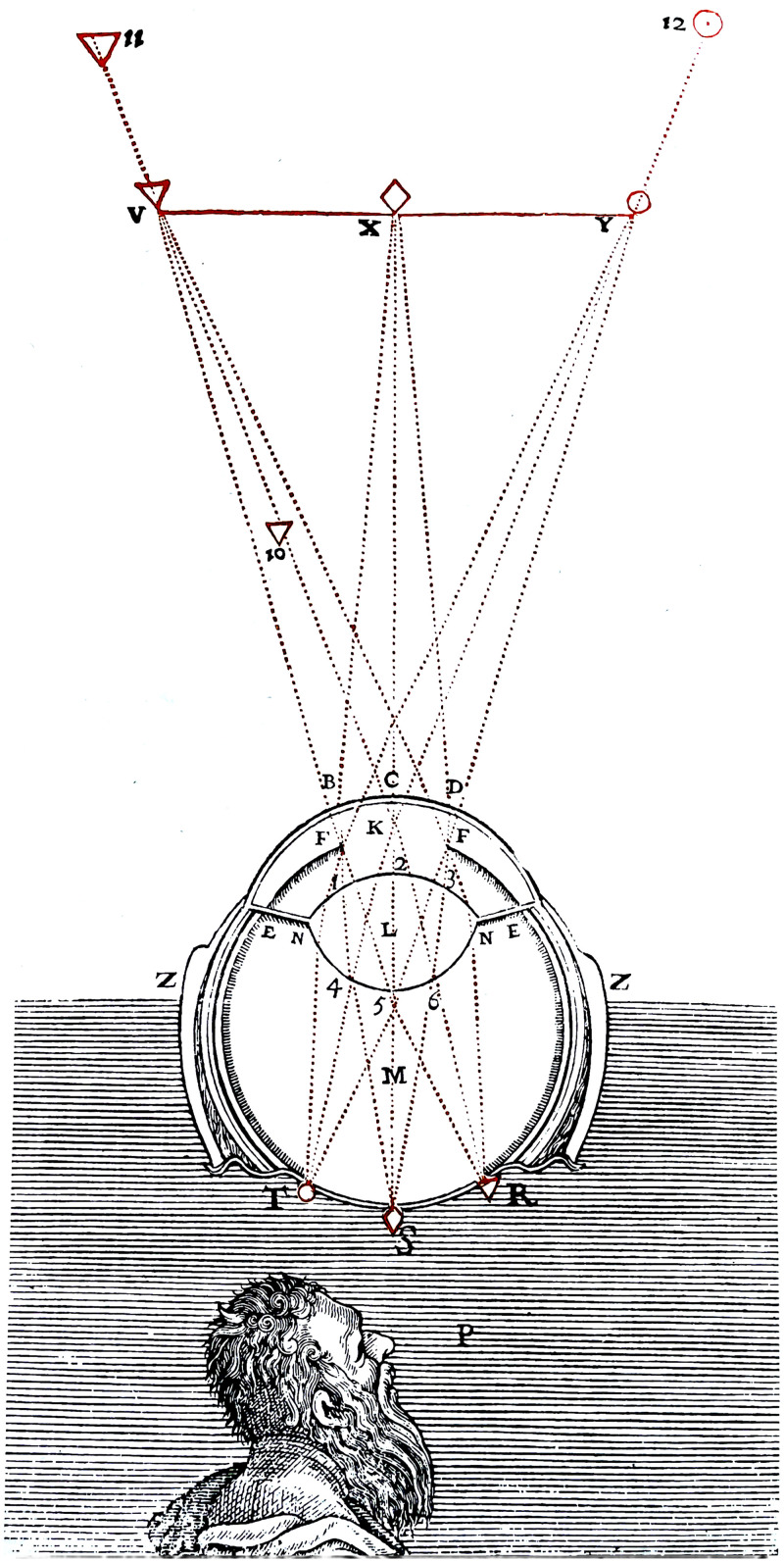
Kepler's optics as illustrated in Rene Descartes’ *La Dioptrique*.

**Figure 5.**
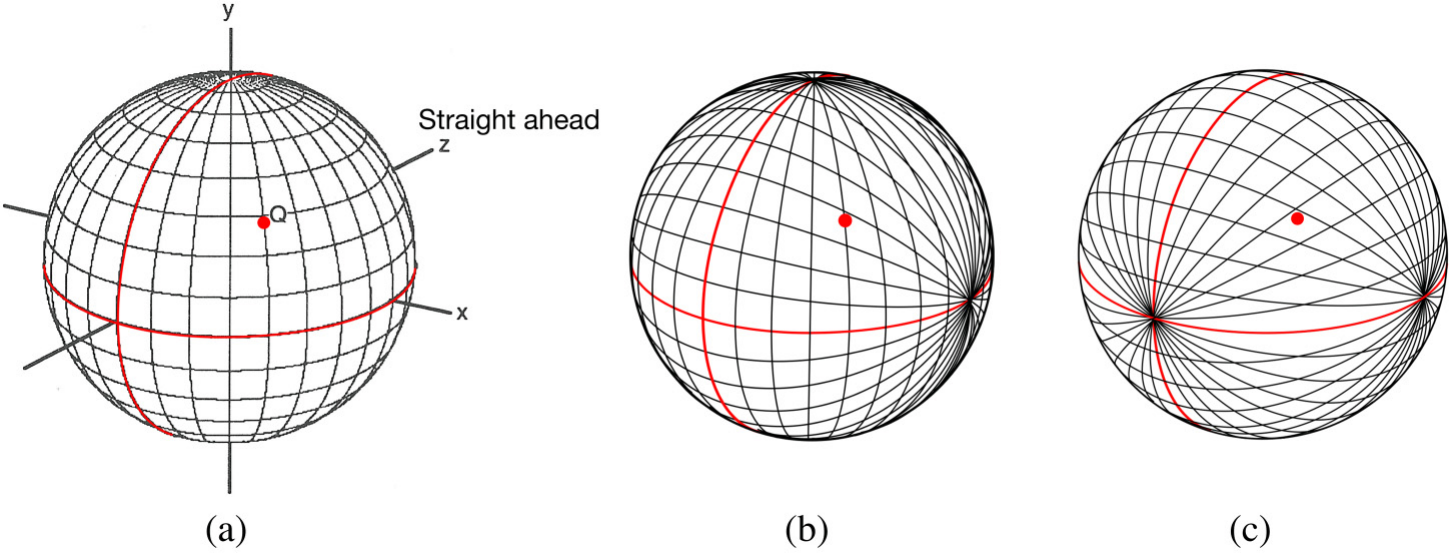
(a) In a “longitudinal/latitudinal” coordinate system, point Q is specified in terms of its azimuth angle (how many degrees east or west of the Greenwich meridian) and its elevation from the equatorial plane (how many degrees north or south). (b) In a “longitudinal/longitudinal” coordinate system, great circles pass through the “east/west” and “north/south poles” of the projection sphere. (c) In an alternative “longitudinal/longitudinal” coordinate system, great circles pass through the “east/west” and “near/far poles” of the projection sphere.

Let us first consider the projection of a point in the surrounding world onto a single, approximately spherical eyeball, as beautifully illustrated in Rene Descartes's *La Dioptrique* (Figure 4). The retinal position of the point specifies *visual direction* with respect to the eyeball—that is, its oculocentric location. Let us assume that there are orthogonal axes in the eye to specify what is horizontal and what is vertical. Visual direction can then be determined by the angles of elevation and azimuth of that point but the sizes of those angles also depend on the particular coordinate system used. Take, for example, the geographical location of places on the earth's surface. In this case, the convention is to use what we can call a “longitudinal/latitudinal” coordinate system (Howard & Rogers, 1995) where the lines of longitude are great circles passing through the north and south poles, and lines of latitude are lines of equal elevation with respect to the equatorial plane (Figure 5a). By convention, longitude is specified with respect to the Greenwich meridian—the line of longitude passing through Greenwich in the UK.

Hence the geographical coordinates of Kyiv are 30.5E° azimuth and 50.5N° elevation (Point Q in Figure 5a). But this is not the only possible way of describing a point in space using spherical coordinates, as Howard and Rogers (1995)^2^ have pointed out. The choice of the coordinate system becomes crucially important for answering the question of whether there are, or are not, disparities between features in the optic arrays from two spatially separated vantage points, and also for the distinction between “horizontal” and “vertical” disparities.

The “longitudinal/latitudinal” coordinate system of geographical position in Figure 5a is just one of a number of coordinate systems that specify the locations of points in surrounding space. For human binocular vision, the “longitudinal/latitudinal” coordinate system has the advantage that there are no changes to the vertical disparities of a point when the eyes make *vergence* eye movements because the coordinate system has a vertical (i.e., north–south) axis of rotational symmetry. An interesting alternative is a “longitudinal/longitudinal” coordinate system with great circles that pass through the “east/west” and “north/south” poles of the projection sphere (Figure 5b). The horizon plane (horizontal red circle) is defined by a particular great circle that passes through the “east/west” poles. In this coordinate system, all horizontal lines that are straight and parallel (in a straight-ahead direction) project to great circles that pass through these “east/west” poles (Rogers & Brecher, 2007). All vertical lines that are straight and parallel (i.e., lines that are perpendicular to the horizon plane) also project great circles that, in this case, pass through the “north/south” poles.

There is, however, a major problem when attempting to measure both horizontal and vertical disparities using any coordinate system that is based on *retinal* coordinates: we have eyeballs that can move—horizontally, vertically, and torsionally. In other words, the disparities of individual points are not *invariant* with eye movements and misalignments of the eyes. However, it is important to note that the world we live in is made up of surfaces rather than isolated points, so perhaps there could be invariances in the property of surfaces, as I will argue later.

## Optic Arrays and Helmholtz’s “Celestial Sphere”

**Figure 6.**
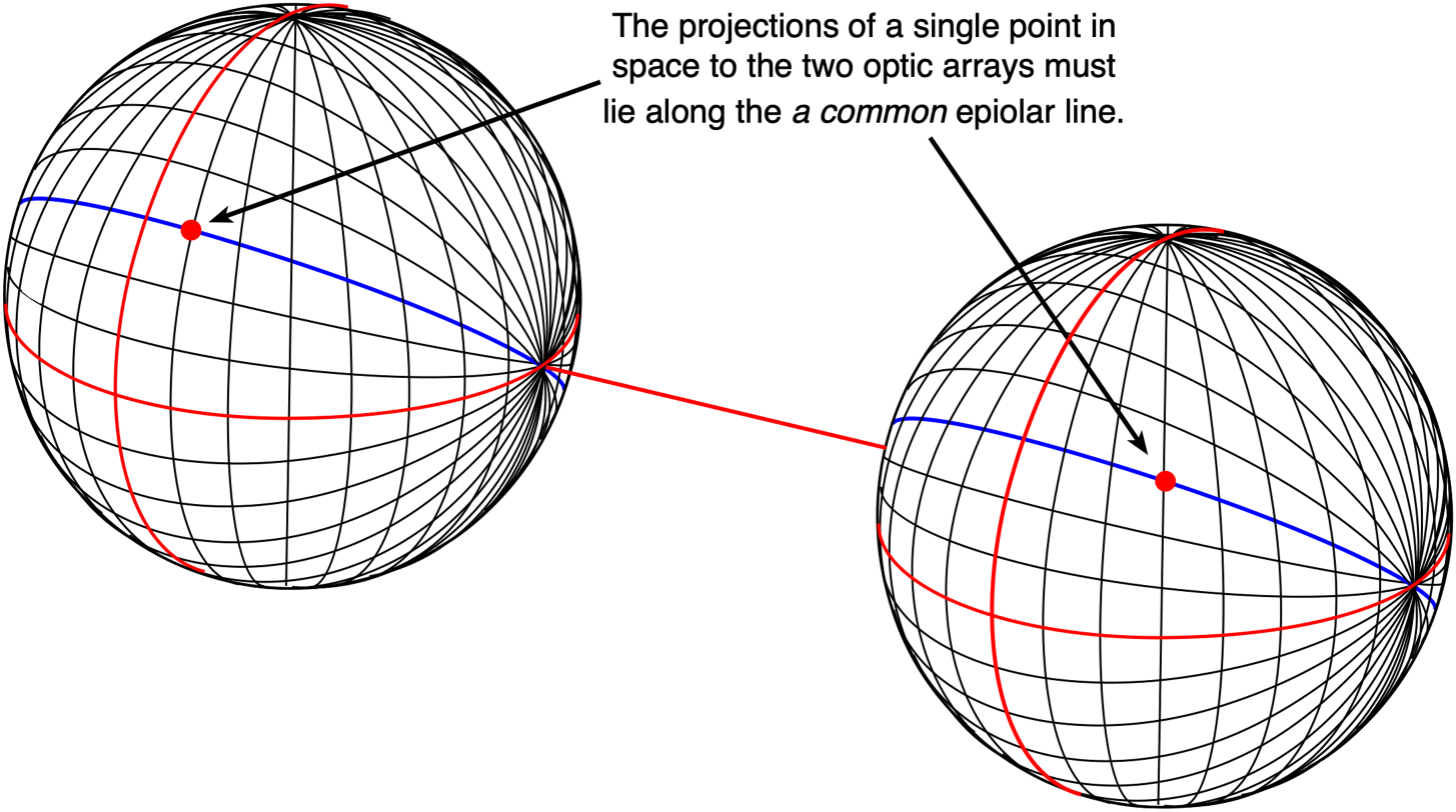
The projections of a single point anywhere in space onto the two “celestial spheres” must lie on great circle lines that define an epipolar plane.

**Figure 7.**
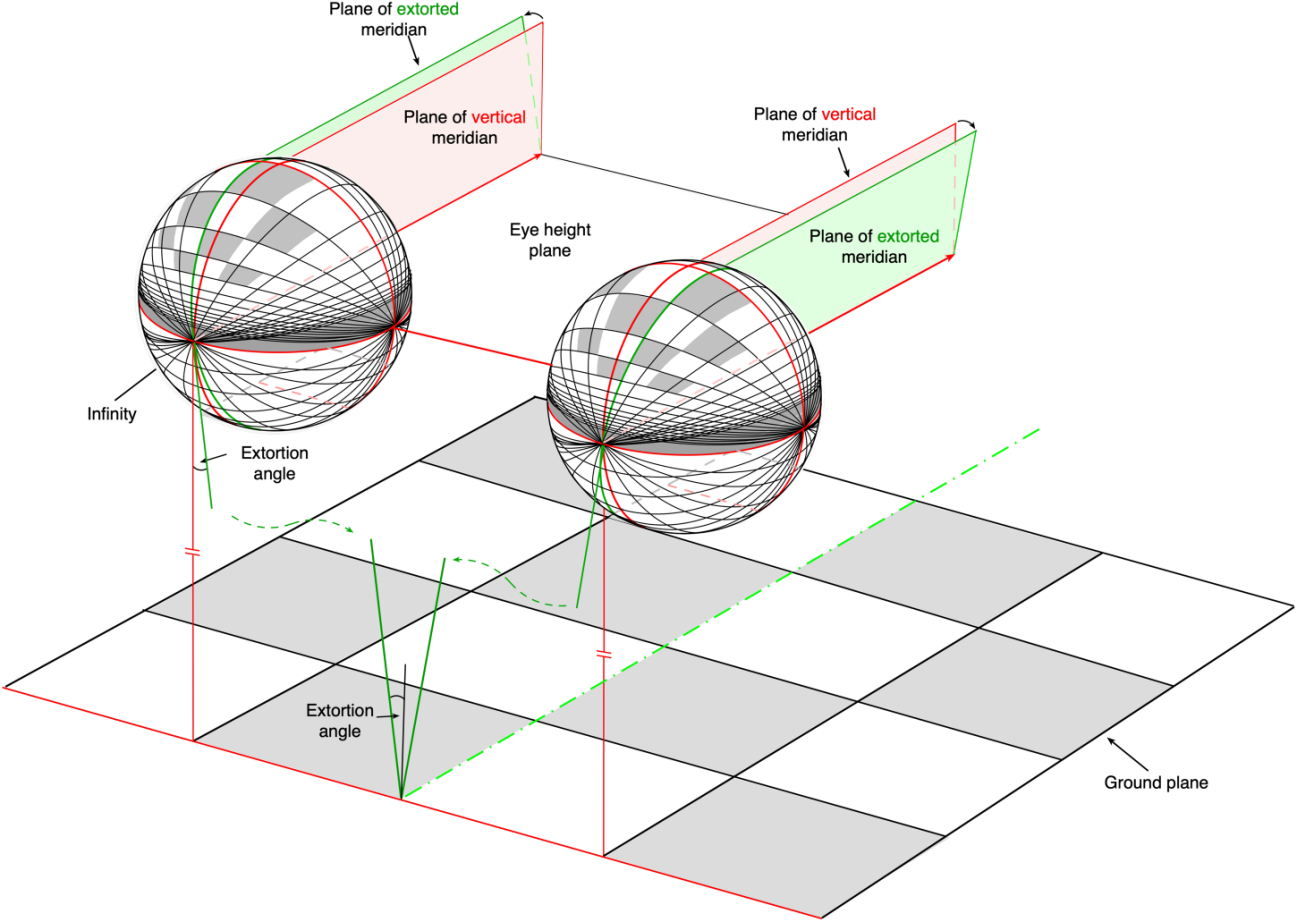
The projection of a checkerboard ground plane surface onto two, spatially separated spherical surfaces with longitudinal/longitudinal coordinates. The *left-to-right* contours of each of the checks on the ground plane surface project to *corresponding* “east/west*”* great circles on each of the celestial spheres. On the other hand, the *near-far* contours of each of the checks project to *corresponding* great circles with respect to the *extorted* meridians of the center line (shown in green).

**Figure 8.**
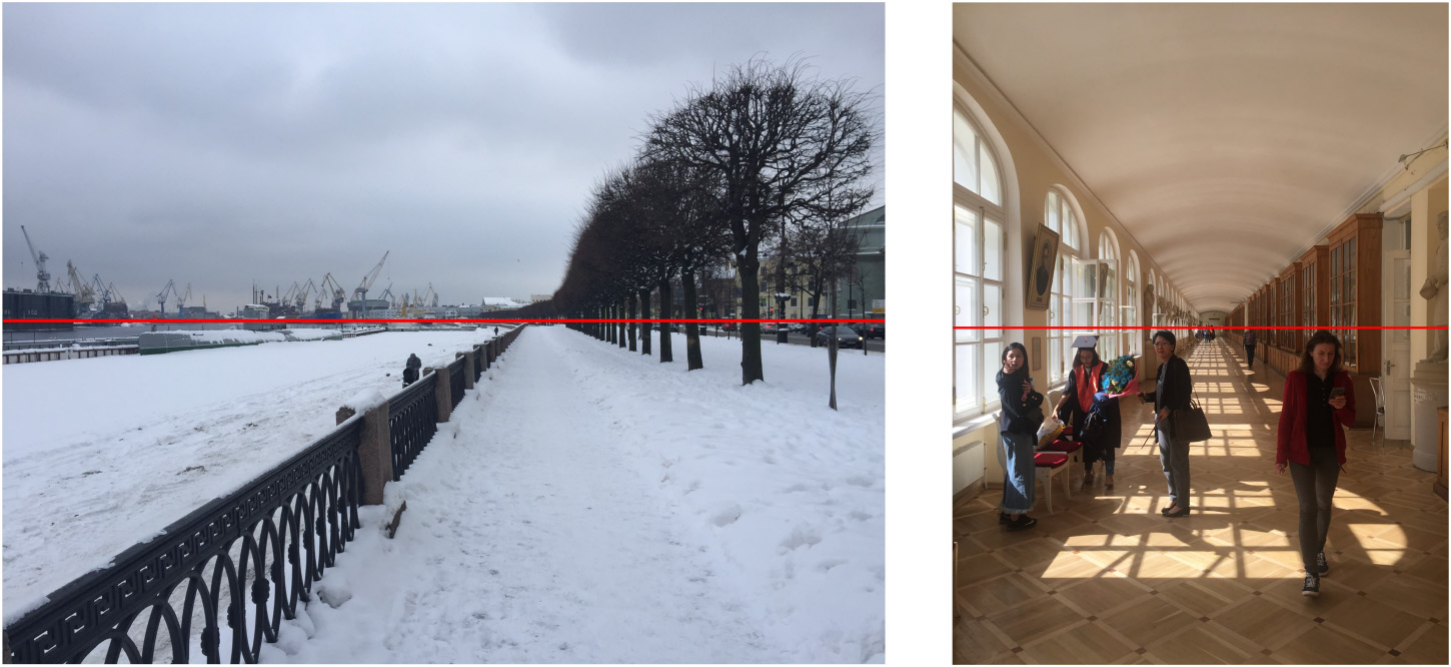
(a) An illustration of Sedgwick's “horizon ratio.” The red line of the visible horizon “cuts” the row of trees on the right-hand side of the photo by the same above-to-below ratio irrespective of the distance of the trees from the observer (Rogers, 2020). (b) All of the people in this scene are shorter than the photographer because their eye heights are all below the line of the visible horizon.

In order to understand the concept of binocular disparity better, it is necessary to take a step backward and consider *why* there are differences between the two retinal images (Rogers, 2007, 2009). Disparate retinal images are a consequence of viewing the surrounding world from two spatially separated locations or *vantage points* (let us forget about the eyes for a moment). To do this we need to consider the differences in the *optic arrays* at two spatially separated locations. The optic array concept (Gibson, 1966, 1979) is important, not just because it is physically and logically prior to the formation of retinal images (Rogers, 2007), but also because it is a description that is independent of any particular visual system, either biological^3^ or man-made. An optic array is a description of the pattern of light reaching a particular vantage point, expressed in angular units. As a consequence, it is not surprising that we express binocular disparities (both “horizontal” and “vertical”) in *angular units*—degrees or minutes of arc, rather than the metric differences between points in the images reaching the two eyes. In order to visualize the properties of an optic array, it is useful to project the angles of the optic array onto a spherical surface surrounding the vantage point, since this preserves the optic array properties. For example, the magnitude of the separation between two different angular directions exactly maps onto their spatial separation on the spherical surface. In the third volume of his *Handbuch der physiologischen optik*, Helmholtz (1867) made the distinction between the *visual globe* (Sehfeld) of the eye (which moves with the eye) and the field of fixation or *celestial sphere* (Blickfeld) which remains fixed (Rogers & Brecher, 2007). Figures 6, 7, and 8 use Helmholtz's idea of a *celestial sphere* to reveal the properties of the optic array.

Helmholtz wrote:

… I prefer to consider the two surfaces (the visual globe and the field of fixation or celestial sphere) that are outside the eye rather than the retina and the retinal image, because the former are a more correct expression of our actual consciousness, and because by directly referring all places to the two spherical fields we avoid the ambiguity that is responsible for so much that is erroneous here; whereas when we speak of knowing the positions of objects by the places on the retina that are affected by them, we seem to imply that we are aware of the retina and know something about its dimensions and extent. (Helmholtz, 1910, p. 166)

Consider the optic arrays using a “longitudinal/longitudinal” coordinate system at two spatially separated vantage points (Figure 6). Any point in space will project to the two *corresponding* great circles^4^ that pass through the “east/west” poles in each of the two optic arrays. These lines are referred to as “epipolar lines” (Hartley & Zisserman, 2004). This means that there are no *vertical* differences or disparities between the two projections of the same point with respect to the “east/west” epipolar lines—that is, they lie on the *same* elevated plane—an epipolar plane. It has been argued that this fact of geometry makes it easier to identify the *corresponding* points in the two eyes because those points are constrained to lie along the same epipolar line. However, the *epipolar constraint* (Frisby & Stone, 2010; Hartley & Zisserman, 2004) only works for a pair of optic arrays (or eyes) with parallel axes (i.e., directed at infinity) and is not the case if the two eyes converge (unless the visual system has independent information about eye position).

## Optic Arrays and Ecological Optics

If we consider an optic array conception of binocular disparity, “horizontal” is no longer a problem—it is defined by the horizontal plane of the ground surface on which we live, and vertical refers to the direction of gravity (Sprague et al., 2015). The two terms are necessarily linked—once you have established the horizontal ground plane, the direction of gravitational vertical follows and, conversely, once the direction of gravity is established, the specification of the ground plane follows (Rogers, 2020). Unlike the “horizontal” and “vertical” axes of retinal image descriptions, there is no arbitrariness and no dependence on the state of the animal's eyes.^5^ However, the specification of disparities in spherical coordinates of the optic arrays does not solve the problem of how we should measure disparities because this depends on the particular choice of the coordinate system. In what follows, I have chosen to consider the optic array disparities that are created by two particular surfaces in the world: (i) the ground plane surface (i.e., the horizontal) and (ii) frontal surfaces (i.e., surfaces that lie in a vertical^6^ plane) since these represent the primary surfaces for an ecological analysis of binocular stereopsis (Michaels & Carello, 1981).

## The Projection of the Ground Plane Surface

There is one particular coordinate system that has some interesting properties for an ecological analysis of binocular vision—a “longitudinal/longitudinal” coordinate system with great circles that pass through the “east/west” and “near/far” poles of the celestial sphere (Figures 5c and 7). All lines on the ground plane that are straight and parallel to the horizon, that is, horizontal lines, project to great circles passing through the common “east/west” poles (epipolar lines). Figure 7 also reveals that a line on the *ground plane* from infinity (in the straight-ahead direction) to under the observer's feet (the dashed green center line on the ground plane) does *not* project to corresponding great circles in the two celestial spheres. Instead, that dashed green center line on the ground plane projects to the two *extorted* meridians—the *green* great circles on the two “celestial spheres” in Figure 7.

This characteristic was first described by Hermann von Helmholtz in Volume 3 of his *Physiological optics* (Helmholtz, 1910, pp. 426–427). One can think of the *extortion angle* created by the ground plane center line as being a “*virtual* convergence angle”^7^ of the eyes.

Consider a grey and white checkerboard located on the ground plane under the observer in Figure 7. The projections of the *left-to-right* contours (i.e., the horizontal contours) of each of the corresponding grey or white checks on the ground plane fall on the *corresponding* great circles passing through the “east/west” poles in the two optic arrays. What about the *horizontal* differences between the projections of the *near-far* contours of the corresponding checks in the two optic arrays? With respect to the celestial spheres, each of the *near-far* contours on the ground plane projects to two different and therefore *noncorresponding* great circles in the two “celestial spheres,” as shown in Figure 7. However, with respect to the two *extorted* great circles (shown in green), the near-far contours of each of the checks lie on *corresponding* near-far meridians. In other words, there are neither horizontal nor vertical differences in the locations of the projected contours of each of the ground plane checks when referred to (i) the common “east/west” poles of the two optic arrays and (ii) the two *extorted* great circles passing through the “near/far” poles.

Note that the angular extent of the *extorted meridians* in the two optic arrays is an *inverse* function of the vertical distance between the vantage points of the optic arrays and the ground plane. The angular extortion is maximal occurs when that vertical distance or eye height is small and approaches zero as the eye height tends to infinity. For a person with an eye height of ∼165 cm and an interocular separation of 6.25 cm, the angle of extortion is ±2.17°, as calculated by Helmholtz 150 years ago. (Note that the extortion angle has been deliberately exaggerated in Figure 7).

However, it is important to stress that the preceding analysis is based on theoretical calculations using a single eye height and a single interocular separation. In practice, Cooper et al. (2011) have shown whilst the *average* shear angle between the extorted meridians of their observers was similar to the predicted shear angle based on the *average* eye height/separation, there was not a simple relationship between a given *individual's* shear angle and their *individual* eye height/interocular separation. Moreover, those authors found that the empirically determined shear angle between the extorted meridians did not remain constant with the angle of declination from the straight ahead (i.e., the green great circles in Figure 7) but rather that the shear angle varied in a curvilinear fashion (at least for some observers). If this were generally true, it would be inconsistent with the idea of optimizing the extorted meridians to near-far lines on the horizontal ground plane.

## Extorted Meridians

Is there any reason to think that the human (or another animal's) visual system makes use of this feature of the binocular optic arrays? Whilst no one would want to argue that our visual system has any explicit “knowledge” of projective geometry, it is possible that the visual system makes use of these particular geometric properties as a result of everyday experience. Eye height *increases* slowly over the first 20 years of our lives and, as a consequence, the degree of extortion of the near/far meridians in the two eyes created by features on the ground plane surface, slowly *decreases*. Given the importance of eye height for a variety of perceptual and locomotor tasks (Sedgwick, 1973, 1986, 2021), it would not be too surprising to think that our visual system could be capable of adjusting or updating the locations of those extorted meridians over time. This would have the effect of altering what are traditionally regarded as “corresponding points” in the retinas of the two eyes. And this was what Helmholtz proposed over 150 years ago. If true, the projections of features on a flat, ground plane surface would never result in any differences between two optic array projections.

There is empirical evidence to support this idea. In adult observers, the greatest sensitivity to small changes in the inclination of “close-to-vertical” surfaces is not around the true vertical but rather around a surface that is inclined *backward*, depending on the viewing distance (Breitmeyer et al., 1976; Cogan, 1979; Ledgeway & Rogers, 1999; Nakayama, 1977). In addition, Cooper and Pettigrew (1979) have shown electrophysiologically that the corresponding vertical meridians of owls and cats are extorted by an amount that is consistent with the eye height of these particular animals. Indirect evidence for the importance of the ground plane has been reported by Hollard and Delius (1982). They found that whereas humans take *longer* to identify spatial patterns as a function of the rotation angle between the original and test patterns, pigeons were equally fast in identifying all orientations of the pattern. When pigeons are flying, they look *down* toward the ground and as a consequence, a particular object can appear at *any* orientation. Hence pigeons need to be able to recognize objects irrespective of their orientation. For humans and other terrestrial animals who typically look out horizontally over the surrounding ground plane, objects (including trees and other animals) tend to have a particular, upright orientation because of gravity. In this respect, young babies seem to have more in common with pigeons in that they do not show the usual *inversion* effect, that is, longer recognition times for inverted faces in adults (e.g., Rossion & Gauthier, 2002). This finding would be consistent with the fact that young babies typically spend a significant proportion of their time lying on their backs where faces can appear at any orientation.

There is also anecdotal evidence that human observers are very sensitive to changes in eye height. When discussing the details of an experiment we designed to measure observers’ sensitivity to differences in simulated eye height (Rogers & Cherniavskaia, 2019), my colleague Hannah Smithson remarked that she noticed the world initially looked different when she put on her high-heeled^8^ shoes. Our experimental results showed that the sensitivity of binocular observers to differences in eye height was around ±2.5 cm, which corresponds to an angular difference in the extorted meridians (or “virtual convergence angle”) of ∼ 2% or 2 arc min.

Having access to eye height information is potentially important because it allows the distance from the observer along the ground plane to be determined by simple geometry using the angle of declination from the visible horizon. In addition, Sedgwick (1973, 1986, 2021) has argued that the visible horizon is important for judging the relative size of objects at different distances using what he referred to as the “horizon ratio.” Figure 8a shows that that all the trees (which are of similar height) are “cut” by the horizon line with the same above-to-below ratio irrespective of their distances from the observer. Sedgwick also pointed out that the height at which the objects are “cut” by the horizon line is equal to the observer's eye height. The significance of Sedgwick's geometric analysis lies in the fact that there is no need for size to be “scaled” by some explicit estimate of distance. For example, the easiest way to find out whether a person standing close to you is taller or shorter is to see whether their eye height is above or below the line of the visible horizon (Figure 8b).

## The Projection of Frontal Surfaces

### Monocular Images of a Frontal Surface

**Figure 9.**
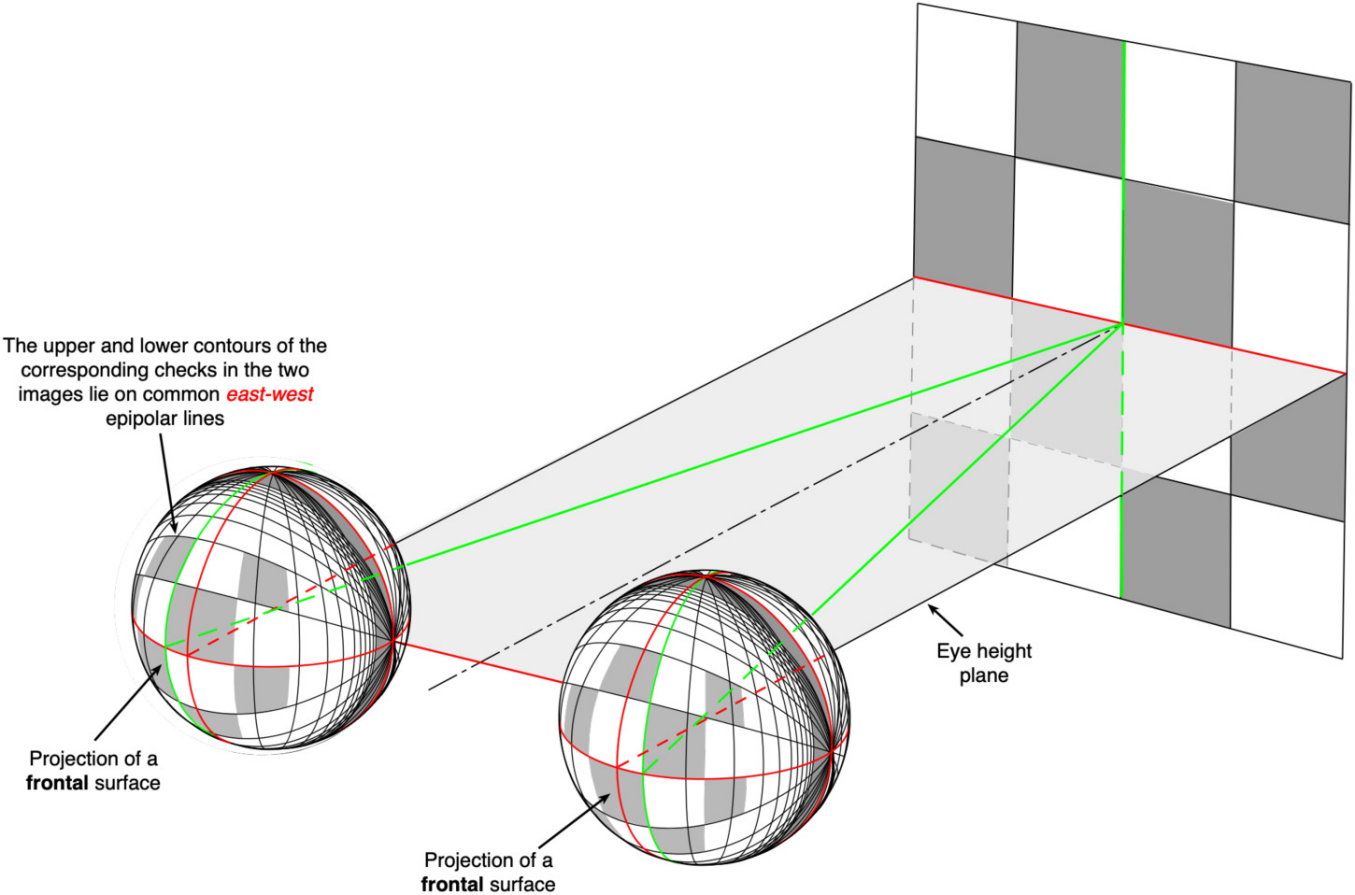
The projection of a nearby checkerboard lying in a gravitational frontal plane onto two, spatially separated spherical surfaces with longitudinal/longitudinal coordinates. The upper and lower contours of each of the checks project to corresponding “east/west” great circles. On the other hand, the left- and right-hand contours of each of the checks do **not** project to corresponding “east/west” great circles. The magnitude of the difference varies with viewing distance. For example, the green vertical line on a nearby frontal surface projects to two disparate great circles in the two optic arrays, shown in green.^19^

**Figure 10.**
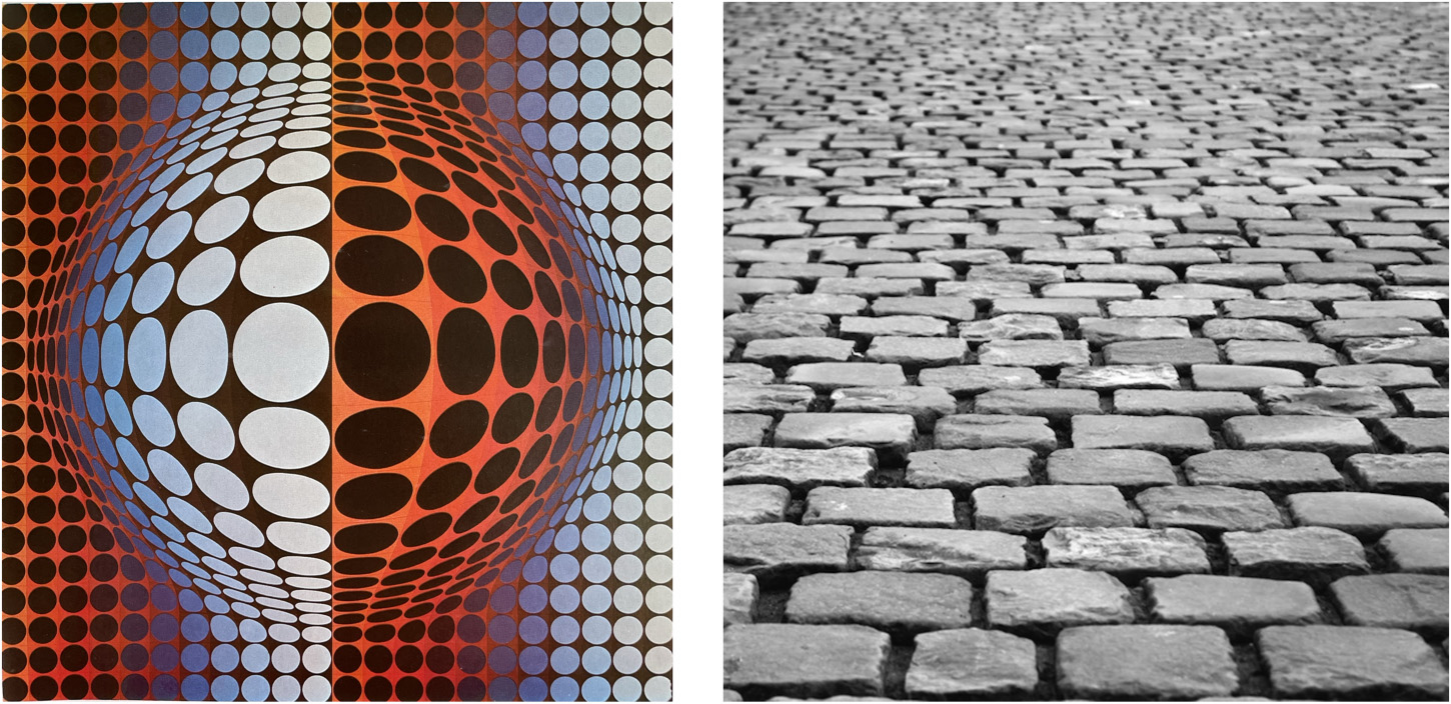
(a) Vasarely’s 1968 artwork *Vega-Wa-3*. (b) A ground plane surface of cobbles that illustrates the vertical gradients of horizontal size and foreshortening.

So far, we have considered the properties of a binocularly viewed *ground plane* surface because this represents the primary reference surface for all terrestrial animals. In this section, we consider the associated reference frame—the gravitationally defined *frontal plane*—that is orthogonal to the ground plane. Figure 9 shows that, in each of the two celestial spheres, the projected sizes of the checks in a regular checkerboard *decrease* in both their width and height with increasing eccentricity from the center of the projection. The geometry of these size changes is straightforward. Along the *vertical* meridian of that frontal surface, the angular *width* of a small check varies as a cosine function of eccentricity (ɛ; i.e., its *elevation* from the horizontal plane) because the *distance* from the vantage point to a particular check increases as 1/cosine function of vertical eccentricity, thereby creating the *size* gradient. Along the same vertical meridian, the angular *height* of a small check on a frontal surface varies as a *cosine squared* function of vertical eccentricity (ɛ) as a result of two factors (i) the *distance* from the vantage point to a particular check which increases as 1/cosine function of vertical eccentricity (i.e., elevation) and (ii) the *slant* of the same small check which increases as a cosine function of vertical eccentricity—creating what is referred to as a *foreshortening gradient*. The opposite pattern of angular size changes can be seen along the horizontal meridian (Figure 9). The pattern of size changes along the vertical and horizontal meridians is beautifully captured in Victor Vasarely's 1968 artwork *Vega-Wa-3* (Figure 10a). Moreover, exactly the same projective geometry applies to a ground plane surface as it does to a frontal plane surface.

The (horizontally restricted) view of a typical ground plane surface shown in Figure 10b illustrates the gradients of size and foreshortening that have been extensively discussed and researched over many years (e.g., Cutting & Millard, 1984). But note that the size and foreshortening gradients in Figure 10b represent only the *one-dimensional* case (the changes in a single direction) rather than the size and foreshortening gradients created by an actual ground plane or frontal surface (Figures 7 and 9). In fact, the actual patterns of size and foreshortening changes as a function of eccentricity are identical for both frontal and ground plane surfaces. The only important difference is that in the case of the ground plane, only the upper half of the gradients in Figures 9 and 10a would be available to forward-facing eyes because the location of the maximum height and width of checkerboard squares is located directly below the vantage point. In other words, we are not able to see the continuation of the ground plane extending behind our feet! However, the important point is that the projective geometries of frontal and ground plane surfaces are essentially the same.

### Binocular Images of a Frontal Surface

Figure 9 shows how the horizontal and vertical contours of a checkerboard surface lying in a gravitational frontal plane project to great circles passing through the east–west and north–south poles, respectively, in each of the two optic arrays. Moreover, the upper and lower contours of each of the checks project to *corresponding* east/west meridians in the two projections, that is, they lie in the same epipolar plane (Figure 6). However, the vertical contours of each of the checks do *not* project to corresponding north/south great circles in the two projections. Instead, they are disparate and the magnitude of the disparity will vary as a function of viewing distance—for example, the green vertical contour on a *nearby* frontal surface projects to two *different* great circles in the two optic arrays (shown in green). Note that the properties of the optic arrays created by frontal planes are closely related to the properties of the two optic arrays created by the ground plane, as described previously.

There is, however, one important difference. In the case of the ground plane, the *vertical* distance from the two celestial spheres to the ground plane (the “virtual vergence angle”) is relatively constant at any given point in our lives—it corresponds to the eye height of a standing observer. In contrast, the *horizontal* distance from the celestial spheres to a particular frontal plane can vary from a few tens of centimeters to infinity. As a result, the angular sizes of patches on a frontal surface *vary* with the viewing distance of that surface—they are not *invariant* as is the case for ground plane patches. The traditional answer as to how the visual system deals with the variations of angular size with distance is to suggest that angular size has to be scaled by some estimate of viewing distance^9^—that is, *size scaling*. Moreover, it has been assumed that binocular disparities also need to be scaled by some estimate of viewing distance—*disparity scaling*—because disparities also vary with viewing distance (e.g., Howard & Rogers, 1995; Vishwanath, 2014; but see Linton, 2021).

### The Inverse Square law and Disparity Scaling

An analysis of the underlying geometry in these situations shows that the binocular disparities created by a 3D object or surface lying in, or close to, a frontal plane vary with the reciprocal of the square of the viewing distance (*D*)—that is, the *inverse square law* (Howard & Rogers, 1995, p. 37).

To a first (small angle) approximation:

$$\begin{array}{rl} \mathrm{Disparity} & =\frac{\mathrm{IOD}\times d}{D^{2}}\\ ∴\mathrm{Depth}\;\;d & =\frac{\mathrm{Disparity}\times D^{2}}{\mathrm{IOD}} \end{array}$$

where IOD is the interocular distance, *d* is the depth of the object, and *D is the* distance from the observer.

However, for the case of *binocular* viewing of frontal surfaces, there is an alternative to disparity scaling that is *invariant* with distance. Rogers and Bradshaw (1993) showed that the horizontal size ratio^10^ (HSR) of a small patch on a frontal surface is equal to the square of the vertical size ratio (VSR) of the same patch: that is, HSR = VSR^2^ (or alternatively, that the ratio HSR/VSR^2^ = 1). This equivalence provides information about whether or not that surface patch lies on a frontal surface. Whilst this equivalence provides information that a particular *patch* lies in a frontal plane, it does not tell us whether an entire surface lies in the same frontal plane.

**Figure 11.**
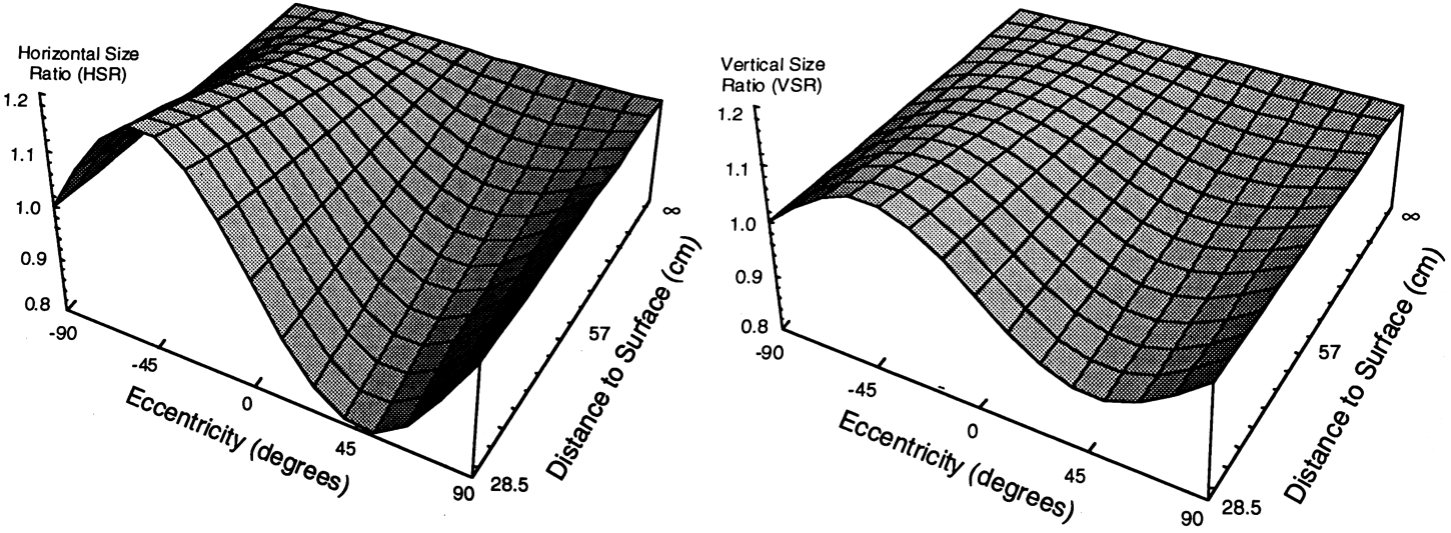
(a) The HSRs as a function of eccentricity for a frontal surface. (b) The VSRs as a function of eccentricity for a frontal surface. (Reproduced from Figures 7.66 and 7.63 of Howard & Rogers, 1995). Note that the *gradient* of the HSR as a function of horizontal eccentricity in the central 90 deg is double the *gradient* of the VSR. HSR = horizontal size ratio; VSR = vertical size ratio.

Fortunately, there is an answer. Geometry shows that if a surface is flat and lying in a frontal plane, the horizontal *gradient of the HSR* as a function of eccentricity is exactly *double* the horizontal *gradient of the VSR* as a function of eccentricity (Figure 11a and b). In other words, it does not matter whether particular points in the scene are disparate, or which frame of reference is used to measure those disparities. The 2:1 ratio of the HSR and VSR gradients as a function of eccentricity provides a reliable source of information that could, in principle, allow us to assess whether a surface is flat and lying in a frontal plane. Even if the observer rotates her or his head to the left or right whilst looking at a frontal surface in the same ego-centric direction (with the consequence that the retinal image in one eye is uniformly larger or smaller than that in the other eye), the ratio of the HSR and VSR gradients remains the same. In other words, there is an invariant characteristic of flat frontal surfaces that a binocular visual system could potentially utilize.

The ability to detect whether a frontal surface is flat would be very useful for a human observer but is there any evidence that the human visual system is capable of exploiting this invariance? In 1995, Rogers and Bradshaw designed an experiment to measure the precision and accuracy of observers’ perceptions of frontal surfaces. In particular, they looked at judgments of the horizontal (left-to-right) flatness of a large, binocular, random-texture-covered-surface, as a function of viewing distance. Figure 3b shows that the pattern of horizontal disparities created by a frontal surface in a left-to-right direction is dependent on the viewing distance, that is, the disparities of frontal surfaces vary in their deviations from the V-M circles depending on distance. Hence, the pattern of horizontal disparities created by a frontal surface is *not* invariant. In Rogers and Bradshaw's experiment, observers were asked to continuously vary the left-to-right pattern of horizontal disparities until the depicted surface appeared to lie in a frontal plane. The results of that study showed that observers were able to do the task with both precision and close to 100% constancy over changes in depicted viewing distance from 29 to 228 cm.

These results are suggestive (but not proof) that the visual system might be capable of exploiting the invariance of the ratio of the HSR and VSR gradients. Note that the same invariance is also relevant to judgments of flatness of *ground* plane surfaces in both a left-to-right and a near-far direction. The 2:1 ratio of HSR and VSR gradients captures the general case of the foreshortening and size gradients illustrated in Figure 9b and Vasarely's depiction^11^ of the size changes in Figure 10a. Moreover, it is equally applicable to the projection of frontal surfaces as it is to the projection of the ground plane surface.

## The Invariant Characteristics of Frontal and Ground Plane Surfaces

The preceding discussion has stressed the importance of *ecological* rather than *geometric* optics, in which the two most significant properties of the world that surrounds us are (i) the *ground plane surface*—to which we are “rooted” as terrestrial creatures (Figure 7) and (ii) the features and objects in the world—the trees, animals, and buildings—whose orientations are typically orthogonal to the ground plane surface as a result of *gravity* (Figure 9; Rogers, 2020; Sprague et al., 2015). By basing the analysis of binocular vision and stereopsis in the ecological framework of the ground plane and the orthogonal axis of the gravitational vertical, many of the traditional problems—such as the need to scale binocular disparities by some estimate of viewing distance—seem to disappear. First, the fact that the angle of extortion of the near/far meridians in the two eyes is stable over time means that we have a reliable source of visual information about the observer's eye height above the ground (Sedgwick, 2021). This provides *visual* information about the “*virtual* convergence angle” of the two eyes and thus the distance to that surface. Second, the geometric fact that the gradient of the HSR (as a function of eccentricity) is exactly double the gradient of the *VSR* (as a function of eccentricity) in *frontal* surfaces, also applies to *ground plane surfaces*. In the latter case, the gradient of the HSR in a ground plane surface is exactly double the gradient of the *near-far size ratio*. This equivalence provides information about the *flatness* of the ground plane surface. Third, there is no need to scale disparities by some estimate of the viewing distance. The geometry of the ground plane provides the scale. If the *ground plane* surface represents the starting point of real-world scene analysis, then the deviations of the actual ground plane surface—its bumps, slants, and undulations—are the ecological disparities. And if the *gravitational vertical* represents a second starting point of real-world scene analysis, then the 3D structure of objects that are deviations from the vertical—for example, the 3D structure of faces, animals, and objects—are also the ecological disparities.

## The “Air” and “Ground” Theories of 3D Vision

The overwhelming majority of studies of binocular vision and stereopsis (including my own) have presented observers with stimuli displayed on monitors or screens located in the *frontal plane* of the observer. This includes the large number of studies that have used random dot patterns as stimuli (Frisby, 1979; Frisby & Clatworthy, 1975; Howard & Rogers, 1995; Julesz, 1962, 1971; Rogers & Graham, 1982; Tyler, 1973, 1991b; Tyler & Julesz, 1978). In other words, the depicted surfaces in those experiments are typically viewed as if they were suspended in the air (and often with a dark surround) at a particular distance from the observer. This approach and analysis have been labeled as an “air theory” of 3D vision (Gibson, 1979; Sedgwick, 1986, 2021). In these situations, the only information about the absolute distance of the depicted surface from the observer is provided by the vergence angle of the eyes and the vertical disparities (if the display subtends an angle that is >20°; Rogers & Bradshaw, 1993).

The fact binocular disparities vary with viewing distance according to an inverse square law has been used to support the idea that in order to recover the depth of the object or surface (*d*), binocular disparities need to be *scaled up* by some estimate of the viewing distance (*D*)—the concept of *disparity scaling*. In support of this suggestion, there is clear evidence that the perceived depth of sinusoidally modulated corrugations is (imperfectly) scaled by both the vergence state of the eyes and the vertical disparities (e.g., Bradshaw et al., 1996). Note that the idea of disparity scaling is closely associated with the idea of *size scaling*, since the angular size of an object varies with the reciprocal of the viewing distance (to a first approximation) and therefore also requires scaling (e.g., Gregory, 1966; Rock, 1984). Once again, there is evidence to show that perceived size is (imperfectly) scaled by the vergence state of the eyes (see Linton, 2021). But is the need for both size and disparity scaling merely a consequence of using frontal plane displays in which objects and surfaces are viewed as suspended in space at a particular distance from the observer? In other words, is the need for both size and disparity scaling a consequence of the traditional “air theory” of perception?

What is the alternative? By definition, humans and other terrestrial creatures live close to a ground plane surface and this has been true throughout our evolution as well as during our lifetime. The texture on a ground plane surface creates gradients of angular size which, together with information about the observer's eye height, provides the necessary scale for judging both size and location (Sedgwick, 2021). Hence there is no need to obtain *explicit* information about distance. Gibson's famous plowed field experiment provides good evidence that accurate size judgments (i.e., there is no systematic bias) can be made at distances up to 400 yards (Gibson, 1950). But note that the role and importance of the ground plane is not new. A thousand years ago, the Arabic scholar Ibn al-Haytham proposed a “ground plane” theory of 3D vision (Sabra, 1989), which has been described and analyzed in detail by Sedgwick (2022). In other words, al-Haytham’s “ground plane” theory is actually an “old theory of 3D vision,”^12^ and hence the title of this paper.

Could the ground plane surface provide a basis for judging the depth and layout of the surrounding world as well as for size? As indicated earlier, simple geometry shows that the angular *width* of texture elements on the ground plane surface is an inverse function of viewing distance (to a small angle approximation) which means that there is a near-to-far *gradient of horizontal angular size* of elements from infinity to a point under the observer. In optic array terms, Figure 8 shows that the projected angular *width* of elements on the ground plane surface is a *sinusoidal* function of the *angular declination* from the horizontal. This can be seen in the increasing separation (as a function of angular declination from the horizontal) between the red and green great circles from the near pole (i.e., zero declination at infinity) to a point immediately below the vantage point (i.e., at 90° declination). But note that this function is close to linear from infinity to a point on the ground plane surface that is at a distance in front of the observer that corresponds to the observer's eye height (i.e., a declination angle of 45°). This gradient, together with information about the observer's eye height, means that there is no need to scale angular size by some estimate of viewing distance based on the vergence angle of the eyes or vertical disparities, as has been traditionally assumed. The ground plane itself provides the scale, even for a *monocular* observer. In addition, Linton (2021) has provided convincing experimental evidence that vergence alone does not affect perceived size.

**Figure 12.**
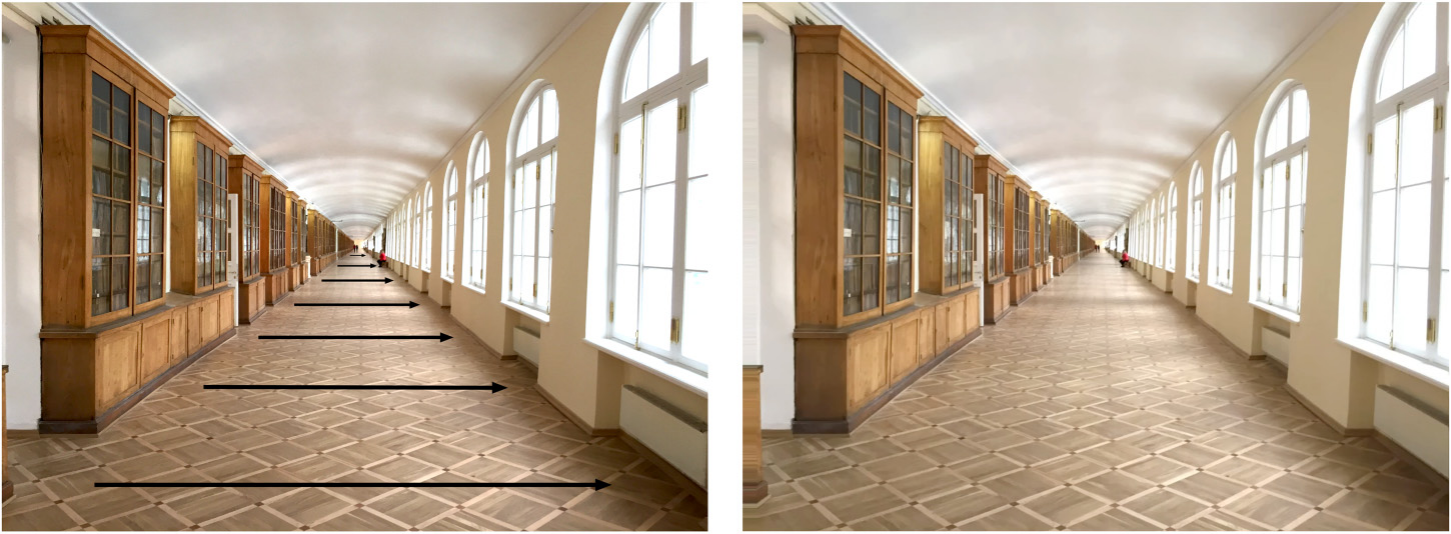
(a) When the observer moves from right to left while fixating on the far end of the corridor, there is a gradient of motion from near to far (i.e., motion perspective) created by the ground plane surface, as shown by the lengths of the arrows. The texture elements of the floor of the corridor also create a gradient of texture from near to far that exactly maps onto the gradient of motion (adapted from Rogers, 2017a). (b) A video showing the gradient of motion created when the observer moves from side to side while fixating a point at the end of the corridor. In optic array terms, both the perspective and the motion gradients can be characterized as *rotations* of the scene around a point at infinity, as can be seen in the movie.

Figure 12a goes further and shows that the *perspective gradient* of horizontal angular width produced by the ground plane surface of a long corridor exactly maps onto the *motion gradient* created when the observer makes a side-to-side movement (when viewing the far end of the corridor)—that is, motion parallax. The movie in Figure 12b shows that the continuous parallax transformation created by an observer moving from side to side can be characterized as a *rotation* of the scene around a point at infinity with respect to the eye. It follows that viewing a scene from *two* stationary, horizontally separated vantage points (i.e., binocular viewing) can also be characterized by a *discreet* (rather than a continuous) rotation of the scene around a point at infinity in the two eyes. In optic array terms, the gradient of the *absolute disparity* created by features on the ground plane can be seen in the *difference* between the locations of the corresponding great circles (shown in *green*) in the two optic arrays of Figure 7. Hence, the perspective gradient, the motion parallax gradient, and the gradient of absolute disparity all provide the same, *complementary* information about the layout of the ground plane, as Gibson (1950) suggested over 70 years ago. This is very different from the traditional view that perspective, motion parallax, and binocular disparities are separate and independent depth “cues” (e.g., Bruce et al., 2003; Howard, 2012; Rock, 1984).

What does this mean for the traditional idea of disparity scaling using some estimate of absolute distance? In real-world perception, there is nearly always a visible ground plane and, as a result, (i) the visible texture on that surface, (ii) observer motion, and (iii) binocular viewing all provide *complementary* information that is needed for perceiving the spatial layout, including the depth, distance, and 3D structure of objects in the scene. If you like, the scale is provided by the ground plane but note that binocular disparities as such do not need to be scaled, any more than the angular subtense of an object needs to be scaled in order to judge relative size. If this is accepted, it raises the question of whether there is any role for the vergence angle of the eyes in the perception of spatial layout in natural scenes (Linton, 2021). On a priori grounds, the use of vergence signals seems unlikely because eye movement signals are both imprecise and labile (adaptable). One hundred and fifty years ago, Helmholtz wrote:

…it might be possible to form a judgment of (absolute distance) by being conscious of the amount of convergence required to direct lines of fixation to the object. But this sensation is quite **unreliable** and **inaccurate**… (Helmholtz, 1910, p. 312, my emphasis).

In terms of practical consequences, I have noticed that observers rarely, if ever, notice when the horizontal positions of a pair of binocular images are misaligned on the display screen. But note that even if there is a lack of alignment (i.e., the vergence state of the two eyes is incorrect), the *gradient of absolute disparity* remains the same. And this is very relevant to our own experimental findings in the 1990s on the role of so-called vertical disparities (Glennerster et al., 1996; Rogers & Bradshaw, 1993, 1995). In those studies, we found that whereas the *perceived depth* in sinusoidally modulated disparity corrugations (in a frontal plane) was very *imperfectly* scaled by changes in vergence angle (∼35% of complete constancy), judgments of the *flatness* of frontal plane surfaces were close to perfect constancy, that is, 100% “scaling”^13^ (Rogers & Bradshaw, 1995).

Disparities may need to be scaled by vergence angle when there is no visible ground plane surface (e.g., looking down from a high building) but scaling is not needed for the judgment of the flatness of frontal plane surfaces when there is a ground plane surface. And these considerations and conclusions are relevant to the “elephant in the room.” As observers, we do not have direct access to the properties of the optic array(s), such as those shown in Figures 7 and 9, but only to the resultant retinal images. However, rotations of the eyes—around either horizontal, vertical, or torsional axes (including the vergence movements of the two eyes)—merely shift the projected images onto different regions of the retina. They do not affect the gradients and the differential structure of features within the image that provide information about the 3D structure of objects and the layout of the surrounding scene (see Koenderink, 1986).

## Further Misconceptions About 3D Vision

### Binocular Disparities or Binocular Transformations?

Whilst it is important to provide an ecological analysis of the binocular disparity concept, many researchers still talk about the spatial *differences* (disparities) between particular *features* in the two optic arrays. In other words, we are still describing the correspondence (or lack of correspondence) between individual *points* or *features* in the two optic arrays or the retinal images (e.g., Read et al., 2009). A quite different approach is to think about how the optic array created by a *surface* (rather than points or features) is *transformed* or *mapped* from one (binocular) optic array to the other (Banks et al., 2004; Michaels & Carello, 1981; Oluk et al., 2022). The simplest example of such a transformation is in the viewing of a surface that lies at an *eccentric location* with respect to the two binocular vantage points: that is, off to one side. Because that surface is closer to one vantage point than the other, the optic arrays from the two vantage points are related by a simple *expansion* transformation.^14^

A second example is the binocular viewing of a surface that is *slanted* or *inclined* with respect to the frontal plane. In this case, the projected images in the two optic arrays are related by a *shear* transformation (Koenderink, 1986). Thinking about binocular vision in terms of the transformations that map the projections of surfaces of one optic array onto the other is not only simpler than a point-by-point correspondence mapping but it also reveals the close similarity between binocular vision and motion parallax as sources of 3D information (Rogers, 1993; Rogers & Graham, 1982). In the binocular case, the transformation is between the two, *discrete* optic arrays from two spatially separated vantage points at a given moment in time, whereas for motion parallax the transformation is based on the *continuous* spatial change of a (single) vantage point over time. In both cases, the mapping is based on the projections of the *surfaces* that surround us in the world, rather than the projections of arbitrary points.

### The Correspondence Problem

The idea of a binocular transformation is also relevant to the so-called “correspondence problem” in binocular stereopsis. To quote Frisby and Stone (2010):

… how can the brain decide which **dot** in the left stereo half is to be matched with a given **dot** in the right stereo half? (my emphasis)

Leaving aside the fact that brains do not *decide* anything, much effort has gone into finding possible computational solutions to the correspondence problem (e.g., Frisby & Stone, 2010; Julesz, 1973; Marr & Poggio, 1976; Pollard et al., 1985) but is it a real problem or merely a consequence of using highly artificial random dot patterns? I would argue that because we live in a world of surfaces that create spatial gradients of texture (rather than a uniform array of isolated points), the visual system never “needs to solve” the correspondence problem—there are no ambiguities or so-called “false matches” of points. Instead, there is a unique transformation between the two binocular arrays created by a visible surface. And this includes the ground plane surface. Recent work on the modeling of binocular stereopsis has shown that correlational algorithms based on the matching of local surface patches are more successful than conventional point-based solutions (Banks et al., 2004; Oluk et al., 2022). It is rather ironic that Bela Julesz's attempt to model the processing of binocular disparities 50 years ago was essentially a correlational algorithm (Julesz, 1971). The ambiguous matching of individual points in the “Keplerian array” is a fiction based on geometric rather than ecological optics. For binocular vision, only the transformation needs to be *extracted*—nothing has to be “matched” and certainly nothing needs to be “fused”^15^ (Michaels & Carello, 1981).

### The Use of the Terms 3D Vision and 3D Perception

We are all familiar with the terms 3D vision and 3D perception not least because these terms are used to define one of the major fields of research in visual perception, as well as the chapter headings in many of our textbooks—for example, “The Many Paths to the Third Dimension” (Rock, 1984, Chapter 3). But what is the 3D vision being compared with? 2D vision? I would like to suggest that there is no such thing. We live in a 3D world and therefore it is not surprising that our perceptual system has evolved to allow us to see the colors, depths, shapes, motions, and layout of objects *within* that 3D world. All of our perceptions (not just visual perception) are necessarily 3D because we live in a 3D world. Moreover, we often confuse 2D (i.e., being able to specify something using just two numbers), with flatness. However, the assessment of the flatness of a surface (including paintings and drawings) needs three numbers to specify the characteristics of the surface as a *flat* structure in a 3D world. Even the simple judgment of *visual direction* in a real-world situation—that is, the direction in which an object lies with respect to where our eyes are pointing—oculocentric location (Figure 4)—involves a 3D judgment of where some object or feature is located in the surrounding 3D world—in other words, it is more than just a direction. And this includes the location of a single point in an otherwise dark room. Even in such an impoverished situation, what we see is a point that lies in a particular 3D location—there might be a degree of uncertainty as to its exact location, but it is not *lacking* in the distance dimension—it is seen somewhere “out there.” For example, the stars in the sky are not seen at some indeterminate distance but rather as lying (erroneously) on some flattened 3D dome-shaped surface above us.

Moreover, this misconception is often accompanied by the assumption that we start with a 2D representation to which we have to *add* the (“missing”) third dimension of “depth.” Where does this misconception come from? Most often, it has been attributed to the fact that the retinal surface is essentially a flat or two-dimensional (2D) surface, which has no third dimension.

For example, in 1984, Irv Rock wrote:

But the human retina is effectively a two-dimensional surface. Thus the **puzzle** of depth perception is how we gain formation about distance, and how we make use of this information to **reconstruct** a three-dimensional perceptual world. (Rock, 1984, p. 53, my emphasis)

Nearly 200 years beforehand, the philosopher George Berkeley (1709) championed a similar idea with the statement on the first page of his book “Towards a New Theory of Vision” that:

…Distance, of it self and immediately, cannot be seen.

However, this is only a problem if we are talking about a world of *points*, rather than the world of surfaces that surround us. The fact that the retina is essentially a 2D surface is completely irrelevant. Perception is about extracting (or picking up) information and there are many different sources of information (including binocular disparities), that the human visual system has been able to exploit over centuries of evolution in order to perceive and explore the surrounding world. Note that even the simplest judgment of whether a particular line in space is straight requires a *mechanism* to extract that property. The fact that a straight line projects to a particular set of receptors in the retina is not sufficient to make a judgment of straightness, it requires a mechanism which may, or may not, give the right answer (Rogers & Naumenko, 2016).

## Summary and Conclusions

The traditional concept of binocular disparity as a difference in the locations of a *point* in the images projected to the two eyes is unsatisfactory because it depends on the particular coordinate system used to measure those differences as well as how the “horizontal” and “vertical” are defined. More generally, 3D vision has often been seen as a difficult problem^16^ for the visual system not just because the retina is a 2D surface but also because of the assumption that both size and retinal disparities need to be scaled by some estimate of viewing distance. On the other hand, if we start with the fact that, as terrestrial creatures, there is nearly always a ground plane surface underneath us, it becomes clear that neither angular sizes nor angular disparities need to be scaled by an estimate of viewing distance. The ground plane coupled with the visible horizon, rather than the vergence angle of the eyes or the pattern of vertical disparities, provides the scale for the layout and location of objects in the surrounding scene. Moreover, the gradients of texture, motion parallax, and absolute disparity all provide us with *complementary* information about that layout and the location of objects. Instead of treating these sources of information as different “cues” and then investigating what happens when we put two of these cues into conflict, it would seem to be more profitable to investigate how we perceive natural scenes in which there are multiple, complementary sources of information (e.g., Glennerster et al., 1996). We can then manipulate the features of the scene in order to discover more about the characteristics of the mechanisms that are used to extract that information.

There is one remaining question: How important are binocular disparities? The popular (mis)conception is that having two eyes, and hence binocular stereopsis, is the most important source of information about the structure and layout of the surrounding world. Until recently, the TV manufacturers chose to label their TVs that use polarising or shutter glasses,^17^ as “3D”—as if regular TVs did not provide the viewer with information about the structure and layout of the depicted scene. In contrast, Patrick Hughes’ *Reverspectives* (Papathomas, 2002; Rogers & Gyani, 2010; Rogers & Hughes, 2023), Jan Koenderink's stereo images of people and faces (Koenderink, 2015), and our own experiments involving viewing an Ames Room binocularly (Rogers, 2021), all suggest that binocular disparities might not be as important as we have previously assumed.

In an experiment that we reported at the 2017 ECVP meeting, we asked observers to view large-field images of a variety of natural, real-world scenes that were presented either: (i) monocularly or stereoscopically; (ii) with or without observer-produced parallax; (iii) synoptically^18^ or stereoscopically (Rogers, 2017). Observers gave ratings of the perceived depth and distance using, as a reference, a rating of 10 for the depth perceived in a pair of static, stereo images. Surprisingly, the average ratings of the amount of perceived depth with *monocular* viewing was only slightly lower—8.9—than for stereoscopic viewing (see also Rogers, 1995). In addition, the average rating of the perceived depth with *synoptic* viewing was only slightly lower than for stereoscopic viewing—9.3—in spite of the fact that there were *no* disparities between the two images. In contrast, viewing the same scenes with zero disparity (i.e., like a painting on a flat surface), perceived depth and distance estimates were halved—5.2—as a result of the conflicting information. But note that they were still seen as 3D scenes. Binocular vision might be of particular help for interacting with the world in the close space around the observer but is perhaps of limited significance for the perception of the layout and structure of the natural world.

## Notes

1. The literal definition of a horopter is a *boundary* (horos) for an *observer* (opter).

2. A similar categorization of different coordinate systems (using a slightly different terminology) was used by Read et al. (2009) for their mathematical analysis of binocular disparities.

3. The majority of species do not have chambered eyes (like the human eye) with an array of receptors onto which the image of the surrounding world is projected. In other words, for those species there are no retinal images.

4. Because the separate optic arrays at the two vantage points have a common “east/west” axis (red line), the elevated great circles in each of the two optic arrays coincide with a common epipolar *plane* between the two vantage points.

5. Optic array disparities can also be thought of as “absolute disparities” or “equivalent vergence angles” (Howard & Rogers, 1995, p. 572)

6. Orthogonal to the ground plane.

7. “Virtual convergence angle” because, unlike the typical vergence movements of the eyes, the eyes do not cycloverge under normal viewing conditions (Howard & Rogers, 1995, p. 422).

8. We nick-named this study the “high-heels experiment.”

9. Typically the vergence state of the two eyes.

10. HSR is the ratio of the horizontal angular substances of the patch between the two eyes. VSR is the ratio of the vertical angular substances of the patch between the two eyes.

11. The upper half of Vasarely's artwork depicts the size and foreshortening changes that start from a point just below the observer's eye.

12. This is, of course, a deliberate play on the words used by George Berkeley (1709) for the title of his book: *“An essay towards a **new** theory of vision”* (my emphasis). See also Vishwanath (2014).

13. I have used quotation marks around “scaling” because it seems likely that no scaling is needed for making frontal plane judgments.

14. A similar *expansion* transformation between the two binocular images is created when an observer rotates her/his head around a vertical axis whilst still looking in the same direction (i.e. in same ego-centric location).

15. Note that we never say that the outputs of different cones in the retina need to be “matched” or “fused” but rather that the cone outputs need to be *compared.* The same language is just as appropriate to the comparison of binocular images.

16. Rock (1984) wrote: “We take the three-dimensional world in which we find ourselves so much for granted that we seldom think of our ability to perceive how far away things are from us and from each other as an achievement calling for explanation. …. But the human retina is effectively a two-dimensional surface.”

17. That are no longer marketed.

18. The synoptic images reaching the two eyes are identical and hence there are no differences or disparities, assuming that the eyes are aligned.

19. Hal Sedgwick (personal communication) pointed out that the longitudinal/longitudinal coordinate framework in Figure 9 is just a rotation of the longitudinal/longitudinal coordinate framework depicted in Figure 7 after rotation around the east–west axis through 90°.
